# Strong, Multi-Scale Heterogeneity in Earth’s Lowermost Mantle

**DOI:** 10.1038/srep18416

**Published:** 2015-12-17

**Authors:** Hrvoje Tkalčić, Mallory Young, Jack B. Muir, D. Rhodri Davies, Maurizio Mattesini

**Affiliations:** 1 Research School of Earth Sciences, The Australian National University, Canberra, Australia; 2 Departamento de Física de la Tierra, Astronomía y Astrofísica I, Universidad Complutense de Madrid, E-28040 Madrid, Spain; 3 Instituto de Geociencias (UCM-CSIC), Facultad de Ciencias Físicas, Plaza de Ciencias 1, E-28040 Madrid, Spain

## Abstract

The core mantle boundary (CMB) separates Earth’s liquid iron outer core from the solid but slowly convecting mantle. The detailed structure and dynamics of the mantle within ~300 km of this interface remain enigmatic: it is a complex region, which exhibits thermal, compositional and phase-related heterogeneity, isolated pockets of partial melt and strong variations in seismic velocity and anisotropy. Nonetheless, characterising the structure of this region is crucial to a better understanding of the mantle’s thermo-chemical evolution and the nature of core-mantle interactions. In this study, we examine the heterogeneity spectrum from a recent P-wave tomographic model, which is based upon trans-dimensional and hierarchical Bayesian imaging. Our tomographic technique avoids explicit model parameterization, smoothing and damping. Spectral analyses reveal a multi-scale wavelength content and a power of heterogeneity that is three times larger than previous estimates. *Inter alia*, the resulting heterogeneity spectrum gives a more complete picture of the lowermost mantle and provides a bridge between the long-wavelength features obtained in global S-wave models and the short-scale dimensions of seismic scatterers. The evidence that we present for strong, multi-scale lowermost mantle heterogeneity has important implications for the nature of lower mantle dynamics and prescribes complex boundary conditions for Earth’s geodynamo.

The lowermost mantle (LMM) is the only region of Earth’s interior that compares to the lithosphere in terms of the strength of seismic heterogeneity. It exhibits thermal, compositional and phase-related heterogeneity (e.g.^1^,^2^), isolated pockets of partial melt (e.g.^3^) and strong variations in seismic velocity and anisotropy (e.g.^4^). Determining the spatial distribution of this heterogeneity, and its thermo-chemical nature, is crucial to an improved understanding of key geodynamical processes, including global mantle convection and the associated variations in heat transfer across the CMB (e.g.^2^,^5^), the behaviour of the magnetic field (e.g.^6^,^7^) and the growth of Earth’s inner core (IC) (e.g.^8^,^9^). Although there is increasing evidence to support a heterogeneous LMM in terms of temperature, chemistry, phase and the resulting seismic velocity structure, the debate into the strength and length-scale of these seismic velocity variations is on-going. This lack of concordance, in part, arises from the parameterisation and regularisation procedures of typical seismological imaging inversion techniques (e.g.^10^,^11^,^12^,^13^). Arbitrary model parameterisation, such as blocks or spherical harmonics, prevent the full utilisation of the information content of the data by rigidly enforcing artificial discontinuities, over-smoothing, or over interpreting. An additional issue is the improper handling of data noise. *Ad hoc* methods of estimating error leave models prone to over-simplification, or worse, over-complexity. Here, we overcome these limitations by inverting for data noise and model parameters simultaneously, and accompany our images of the LMM with maps of the associated standard deviation.

In our previous study^14^, we presented a P-wave tomography model of the LMM and the corresponding uncertainty based on a hierarchical trans-dimensional Bayesian method. We build on that here, by examining the model’s heterogeneity spectrum. To address some of the remaining questions regarding LMM heterogeneity, we produce high-resolution images of the LMM compressional wave velocity structure using the hand-picked data set of PKPab-PKIKP, PKPbc-PKIKP, and PcP-P differential traveltimes of Young *et al*.^14^. By using waveform cross-correlation and differential travel times, the biases associated with event mislocation and lateral heterogeneity in the lithosphere are minimised. The inclusion of PcP-P data helps to isolate IC structure from structure in the LMM. We use a probabilistic, Bayesian inversion scheme (e.g.^15^) to invert for LMM structure and obtain a robust representation of the complexity and amplitude of the P-wave velocity heterogeneity, alongside uncertainty estimates (see methods for further details).

To attest to the impressive capabilities of the trans-dimensional and hierarchical Bayesian inversion scheme, we first design and examine a comprehensive, all-embracing synthetic resolution test to demonstrate the recovery of velocity discontinuities, smooth velocity transitions, structures of varying scales and strengths (see Fig. 1A for the synthetic input model). Unlike the resolution test presented in Young *et al*.^14^, this new test, although retaining the configuration of heterogeneity, now also includes velocity gradients that are comparable to those expected in the LMM. The test goes far beyond the conventional checkerboard tests that are commonly utilized, as it checks not only the ability of the inversion method to recover the coexistence of various heterogeneity scales in the lowermost mantle, but also the gradients in seismic velocity field that might be present at the base of the mantle at the transition from one type of structure to another. The Bayesian method successfully recovers relative perturbation amplitudes and discontinuities where sufficient ray path coverage exists (Fig. 1B,C). To further illustrate the ability of our tomography scheme to recover structural complexities in the LMM, we perform a test with a hemispherical structure on which we superimpose smaller semi-circular features in well-sampled equatorial regions, and larger, horizontally-positioned elliptical features at modestly-sampled, moderate latitudes (Fig. 2). Although the PKPab-PKIKP differential travel time dataset successfully recovers most features, aside from within regions where there is no sampling (elevated standard deviation), it is clear that the addition of PcP-P data helps resolve source-receiver ambiguity. The recovery of both long- and short-scale features is impressive, with the standard deviation map fully in concordance with ray-path coverage (Fig. 1C). This test illustrates that image smearing and distortion are minimal and mostly constrained to the Pacific and Antarctica. Despite the fact that Voronoi cells, which underlie the algorithm, have straight edges, curved lines can be fully retrieved after averaging many differently-orientated straight-sided polygons (see methods for further details).

Our results using the observed data (Fig. 3A) demonstrate that the power of heterogeneity in the LMM has a root-mean-square (RMS) P-wave velocity variation of 0.87%^14^, which is 2–3 times larger than previous global-scale estimates (e.g.^10^,^11^,^13^,^16^). The first-order heterogeneity distribution is generally consistent with other P-wave tomographic models of the lowermost mantle (e.g.^11^,^13^,^16^,^17^), including an agreement on the presence of fast velocities beneath Central America and East Asia and slow velocities beneath south Africa and the southwest Pacific. However, there are also clear discrepancies between our images and those of other studies. Apart from differences in velocity structure in North America, Australia, and Europe, we do not observe a strong spherical harmonic degree 2 component. Such discrepancies could be a result of differing datasets, poorer sampling, the inversion method, and/or data noise. The map of standard deviation (Fig. 3B), unsurprisingly, reveals that the greatest certainty is achieved in areas of best ray-path coverage (Fig. 1C). Posterior probability plots of the data noise and the number of cells (Fig. 3C) reveal the estimated noise in each dataset and the number of Voronoi cells to which the inversion converges.

From visual inspection, the length-scale of heterogeneity ranges from ~600–2400 km (measured at the CMB radius, where 1 angular degree is ~60.7 km). For a more rigorous estimate of the lateral extent of velocity anomalies, we perform a 2-D Fast Fourier Transform (FFT) of the velocity pattern and estimate the power spectrum (Fig. 4A,B). The wavelength is given in degrees and is accompanied by the corresponding length scale (in kilometres at the CMB) of heterogeneity. Note that we also provide the results of our synthetic test featured in Fig. 1A,B, which is designed to attest to the ability of the 2D FFT method to retrieve wavelength content (Fig. 4C). We find that the power of heterogeneity in the model steadily increases from a length-scale of ~350 km and peaks at ~1450 km. However, there is substantial and comparable multi-scale heterogeneity, at length scales of ~500–6000 km, which provides a link between the very short-scale features whose presence has been established through scattering experiments (e.g.^18^) and the long wavelength maps resulting from more traditional tomographic approaches with predetermined parameterizations (e.g.^16^). The power spectra of LMM P-wave velocity models from Della Mora *et al*.^17^ and Soldati *et al*.^13^ (Fig. 4A) are shifted toward longer wavelengths and do not exhibit the significant short wavelength (<10 degree) structural content evident in our trans-dimensional hierarchical model. This confirms our visual inspection. Our resolution tests give us confidence this conclusion is not hampered by the contribution from the areas with a higher standard deviation.

Soldati *et al*.^13^ link the CMB topography with geodynamics to predict seismic travel times. To investigate the effect of CMB topography on our results, we compare tomographic models derived from: 1) PKPab-PKIKP and 2) PcP-P datasets. The two independent datasets result in similar models of harmonic degrees 1 and 2 using the Bayesian hierarchical method of Muir and Tkalčić^19^, illustrating that large scale CMB topography does not have a dominant impact on travel times of these two datasets (Figure S1). In the case that CMB topography had a strong degree 1 or degree 2 signal dominating the velocity heterogeneity, it would generate PKPab-PKIKP and PcP-P differential travel times of opposite sign, resulting in negatively correlated velocity anomalies between the two models, which we do not see. We therefore heuristically argue that CMB topography has only a minor influence on our results. Short scale topography on the other hand could still affect the travel time residuals, but a more complete and high quality coverage of the LMM is needed to reach more definitive conclusions.

By allowing the data content to drive the parameterisation and noise assessment and by forgoing smoothing and damping regularisation, we can be confident that the features displayed in our final model are required by the data and are not an artefact of arbitrary modelling and inversion schemes. The absence of shorter wavelength features in previous global models could either be simply a consequence of the truncation of spherical harmonic expansions (e.g.^20^) or due to the strong smoothing and damping employed by tomographic methods with constant velocity block parameterisation (e.g.^10^,^11^).

The long-wavelength component of LMM heterogeneity has been confirmed in a number of studies over recent decades (e.g.^16^,^21^,^22^). Whilst localized scattering studies (e.g.^18^,^23^,^24^) indicate the presence of short-scale structure, the vast majority of global-scale models remain confined to the >1000 km realm. Mapping small-scale structures globally is key to understanding the role of the LMM in global geodynamics. In particular, the multi-scale heterogeneity wavelength spectrum imaged herein helps to discriminate between two possible end-member scenarios for the nature of LMM dynamics: (i) thermo-chemical ‘piles’, where recent subduction history focuses chemically dense material into two distinct, discontinuous structures at the mantle’s base (the large low shear wave velocity provinces – LLSVPs - imaged beneath Africa and the Pacific^1^), which are stabilised through excess chemical density; and (ii) principally thermal heterogeneity, where LLSVPs beneath Africa and the Pacific are attributed to clusters of plumes, which are connected by long linear ridges (e.g.^2^,^25^). The dominant degree two structure of previous tomography models is regularly cited as firm evidence in support of the former (e.g.^26^), implying a key role for chemical heterogeneity in dictating the form of lowermost mantle dynamics. However, the multi-scale nature of lowermost mantle heterogeneity imaged herein, which lacks the dominant degree two distributions of these previous studies and, significantly, contains fast seismic velocity anomalies inside LLSVP regions, is more consistent with the latter (see^27^ for a detailed review). It is important to emphasize that a thermally dominated lower mantle has implications for the long-term stability of LLSVPs, their potential to act as long-term geochemical reservoirs, the source characteristics of deep-mantle plumes and the origin and morphology of ultra-low-velocity-zones (ULVZs) (e.g.^28^,^29^,^30^).

In addition to thermal and/or chemical heterogeneity, a third electro-structural mechanism can provide an explanation for possible small-scale seismic wave heterogeneities in the LMM. As iron is the most abundant transition-metal element in the mantle^31^, iron-bearing minerals should play a role in the geochemistry and geodynamics of the LMM. Therefore, iron-doping in LMM materials opens up the possibility of pressure-induced electronic spin transitions in magnesiowüstite, ferromagnesian silicate perovskite and post-perovskite phases^32^, which could induce small-scale heterogeneities such as those imaged herein. Although, the high-spin to low-spin transition in the Fe 3d states considerably affect the (Mg_0.83_,Fe_0.17_)O sound velocity by ~15%^32^, it is reasonable to believe that, at LMM conditions, only a subtle temperature-modulated velocity jump can be found due to low magnesiowüstite content (20%) and high-temperature conditions. Thus, the aforementioned electro-structural mechanism can possibly explain why the origin of small-scale heterogeneities remains debated and unaddressed in earlier seismic studies.

Strong small-scale structure in the LMM also has implications for Earth’s core. It could explain large differential travel time residuals of PKP wave travel times, possibly offering an alternative to strong IC anisotropy as an explanation (e.g.^33^). Furthermore, at ~2200 km below the CMB, the liquid outer core (OC) slowly crystalizes into the solid IC at the inner core boundary (ICB). There is increasing evidence from studies of seismic attenuation and velocity that the uppermost inner core (UIC) has more than degree 1 complexity^34^,^35^, which could be explained by geodynamical models where spatially variable heat fluxes across the CMB are mapped onto the UIC through a process of thermochemical convection in the OC (e.g.^8^,^9^; see^36^ for a detailed review). Multi-scale heterogeneity in the LMM would likely create more complex thermochemical convection in the OC, which, in turn, would drive inhomogeneous solidification of the IC, a process that would result in the observed lateral variations in the UIC. Future increases in computational power will enable the application of trans-dimensional, hierarchical Bayesian tomography to the whole mantle, thereby shedding more light on this intriguing problem.

## Methods

This study uses a recently developed trans-dimensional and hierarchical Bayesian imaging technique described in depth by Young *et al*.^14^. The LMM (we tested 250, 300 and 350 km thick layer, and found that a 300 km thick layer results in the most robust models and hyper-parameters and the fastest convergence rate) is partitioned into a mosaic of Voronoi polygons (all points within a Voronoi polygon are closer to the nucleus of that polygon than to any other nucleus) whose size, location, velocity, and number vary throughout the inversion according to the information content of the data (partition modelling). The data used are old and newly collected travel time data sensitive to the LMM and core and collected by waveform cross-correlation. We avoid using data from South Sandwich Island events, however, as they may be influenced by IC structure or strong mantle heterogeneity related to slabs or fragments^37^. Upper mantle structure is accounted for by correcting travel times using the mantle model of Della Mora *et al*.^17^. The forward method involves 1D ray tracing. For each source-receiver path, approximately the same number of equidistant points and their locations along each path sampling the lowermost 300 km of the mantle is determined. The ray path geometry is fixed (it does not change with each new realization of Voronoi cells).

The key advantages of the trans-dimensional hierarchical approach are that the number and distribution of the model parameters are implicitly controlled by the data and that the standard deviation of the data noise (assumed to have a Gaussian distribution) is treated as an unknown in the inversion. In the areas of poor or non-existent sampling, the partition modelling will result in a slower convergence and less stable velocity values and Voronoi cell shapes, which will lead to high uncertainty (standard deviation). Our Bayesian method provides the statistical robustness of Garcia *et al*.^38^ and the multi-scale resolution capabilities of Simons *et al*.^12^ in addition to introducing a novel approach to handling data noise and model parameterisation. As a result, the required complexity of the solution is inferred from the data itself (e.g.^39^), rather than from *ad hoc* uncertainty estimation techniques and arbitrary parameterisations so often utilised by more traditional, linearised inversion techniques. The recovery of a LMM map of P-wave velocity heterogeneity requires over 20,000 CPU hours. Although this is five orders of magnitude greater than the time taken by a traditional, non-linear approach (e.g.^11^), the improved quality of the results and provision of uncertainty estimates fully justify the additional cost. Nonetheless, the immense computational cost of such ensemble inference approaches currently prohibits the inversion for whole-mantle structure.

## Additional Information

**How to cite this article**: Tkalčić, H. *et al*. Strong, Multi-Scale Heterogeneity in Earth,s Lowermost Mantle. *Sci. Rep*. **5**, 18416; doi: 10.1038/srep18416 (2015).

## Supplementary Material

Supplementary Information

## Figures and Tables

**Figure 1:**
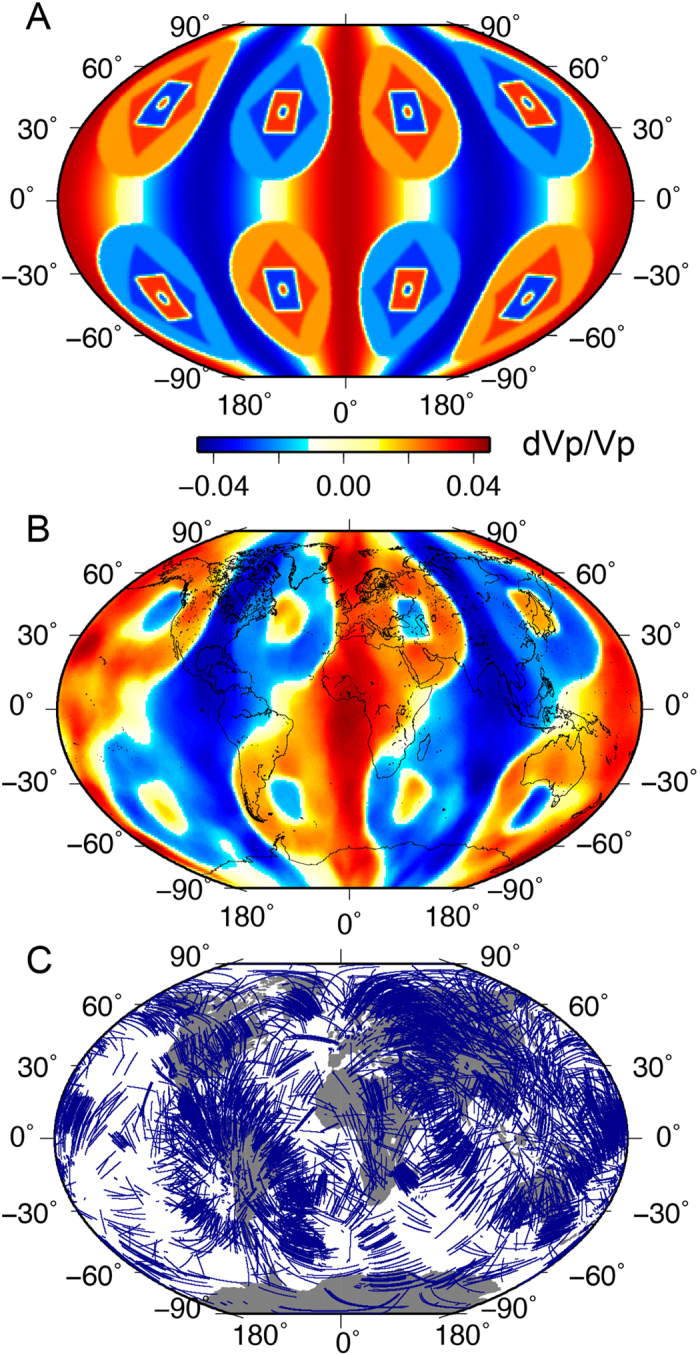
Synthetic test demonstrating resolving power of Bayesian inversion technique using raypath coverage of real data set for the multiplicity of sizes, shapes, gradients and strength of heterogeneity (also see Fig. 3C for the recovery of the power spectrum). (**A**) actual model; (**B**) recovered model after 1 million model iterations; (**C**) ray path coverage of the lowermost 300 km of the mantle for both the synthetic test and the actual model inversion.

**Figure 2:**
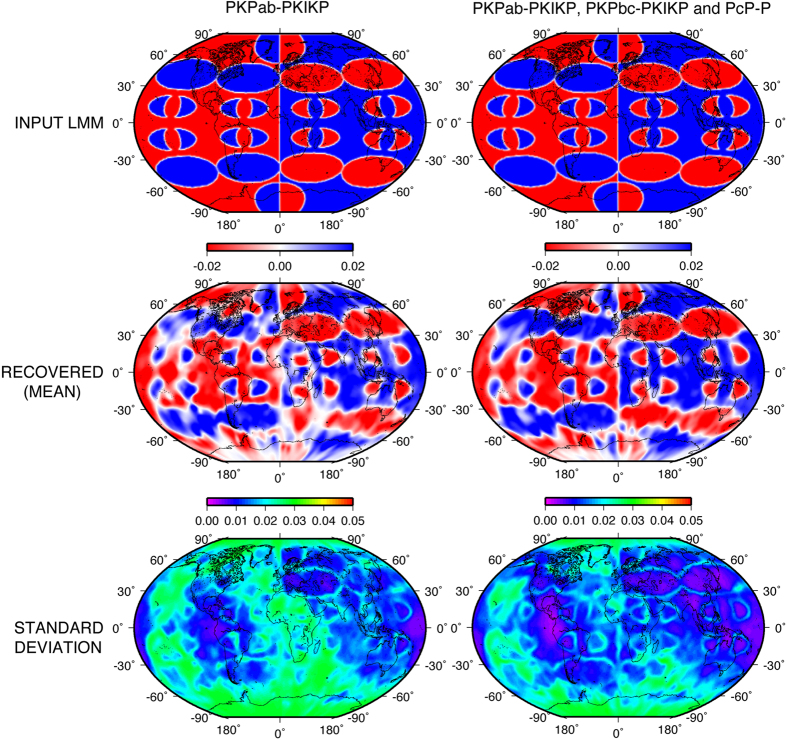
Synthetic test demonstrating the resolving power for the PKPab-PKIKP dataset (left) and combined PKPab-PKIKP with PKPbc-PKIKP and PcP-P datasets (right). Top: actual model. Middle: recovered model (mean). Bottom: standard deviation. Mean and standard deviation maps are computed after 0.7 million model-iterations.

**Figure 3:**
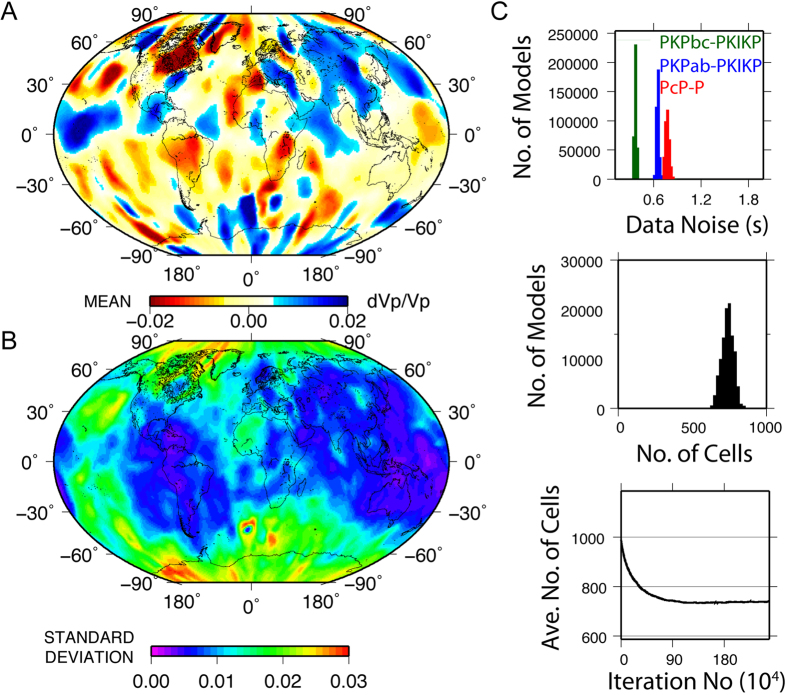
Preferred P-wave velocity model of the LMM. (**A**) P-wave velocity perturbations from the ak135 model^41^ and (**B**) corresponding standard deviation. (**C**) Posterior probability distributions of data noise in seconds for all three types of differential travel time data (top), of the number of Voronoi cells used to describe the model (middle) and the average number of cells as a function of iteration number (bottom). Horizonal axes correspond to the range of priors for data noise and number of Voronoi cells.

**Figure 4:**
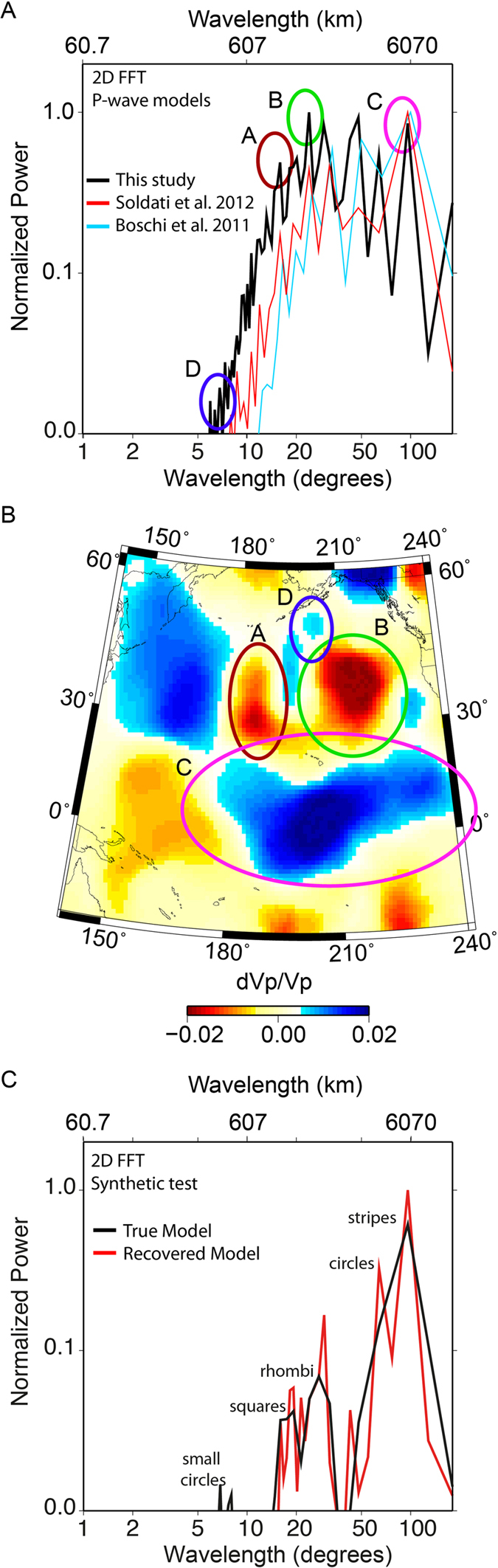
LM-heterogeneity power spectra. (**A**) 2D FFT of final model. (**B**) Demonstration of the versatility of wavelength content for enlarged view of the Pacific region. The corresponding power of the encircled areas is shown in (**A**). (**C**) 2D FFT of true (black) and recovered (red) synthetic models shown in Fig. 1A,B.
