# Supplementary material for: Strong, Multi-Scale Heterogeneity in Earth’s Lowermost Mantle
Source: Sci Rep. 2015 Dec 17;5:18416. doi: 10.1038/srep18416 (PMC4682081; doi:10.1038/srep18416)
Supplement: Supplementary Information [file srep18416-s1.doc]

**Strong, Multi-Scale Heterogeneity in Earth's Lowermost Mantle**

Hrvoje Tkalčić, Mallory Young, Jack B. Muir, D. Rhodri Davies & Maurizio Mattesini

**SUPPLEMENTARY INFORMATION**

Earlier results for global P-wave structure are similar in terms of continental-scale features, but vary widely when it comes to finer scale structure. This suggests that the global models provide only a rough estimate of LMM heterogeneity, leaving small-scale structure largely misinterpreted, or forgotten entirely. In this study we use Bayesian statistics to demonstrate that not only does the LMM indeed contain long-wavelength information, but also a significant amount of shorter-scale structure. The long wavelength features could be a reflection of continental-scale variations in temperature and chemical composition, for example, as would result from subducted slabs or mantle upwelling. The shorter wavelength structure (<1000 km) could indicate the presence of scatterers at the core-mantle boundary, past subduction and partial melt.

Our data-driven approach applies Bayesian statistics to a Monte Carlo Markov chain search of parameter space. This is a probabilistic approach in which the current model is assigned a probability based on the data and on prior information about the known range of acceptable models. Based on Bayes’ Theorem, a mathematical statement of Bayesian theory can be summarized as follows:

*p*(**m** | **d**_obs_ )  *p*(**d**_obs_ | **m**) *p*(**m**)

where *p*(**m** | **d**_obs_ ) is the probability distribution function of the unknown model parameter **m** given the data **d**. The term *p*(**d**_obs_ | **m**) is the likelihood function, which yields the probability of observing data **d** given model **m**. Any prior information about model **m** is represented by the a priori probability distribution *p*(**m**). After a large number of models are sampled via a Markov process, the resulting posterior probability density function becomes the solution to the inverse problem. This is very different to a linear or iterative non-linear optimization approach in that instead of yielding a single value for each unknown model parameter, a complete distribution of values is produced. This allows more complete analysis and understanding of the solution. While, typically, either the mean (as in this study), median, maximum, or standard deviation of the parameter distributions are extracted for interpretation, such calculations merely offer a very concise summary of the final model.


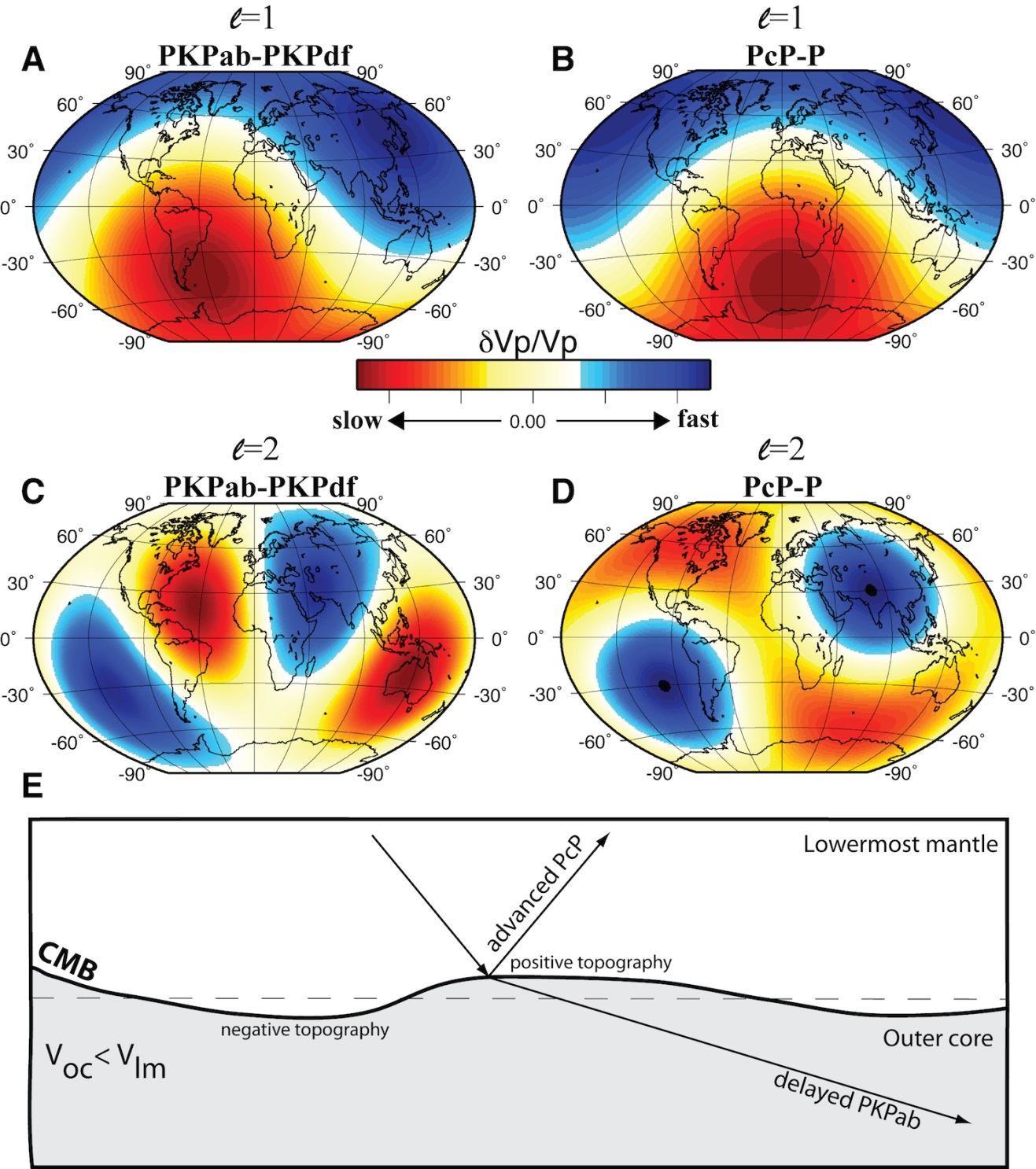


**Fig. S1.** Optimal maximum degree of spherical harmonic expansion is determined through a hierarchical Bayesian inversion to recover various tomographic models for a comparison. (A) Map representation of spherical harmonic degree 1 in the tomographic model derived solely from PKPab-PKIKP travel time data (note that PKIKP is equivalent to PKPdf) and (B) in the tomographic model derived solely from PcP-P travel time data. (C) & (D): same as (A) & (B) but for spherical harmonic degree 2. E) Cartoon showing the interaction of PcP and PKPab waves and the CMB tomography. Positive topography causes the opposite sign of travel time residuals for PcP-P and PKPab-PKIKP, which is not observed in the above maps. The maps were made by Generic Mapping Tools GMT4.1.2 (Wessel, P. & Smith, W. New, improved version of the Generic Mapping Tools, released, *EOS. Trans. AGU* **79**, 579, 1998). http://gmt.soest.hawaii.edu/projects/gmt

Furthermore, we have expanded the tomographic models derived from 1) PKPab-PKIKP and 2) PcP-P differential travel time datasets in terms of spherical harmonics using a Bayesian hierarchical inversion approach (Muir & Tkalčić, A method of spherical harmonic analysis in the geosciences via Bayesian hierarchical inference, *Geophys. J. Int.* **203(2)**, 1164-1171, doi:10.1093/gji/ggv361, 2015). The first dataset contains contribution from the IC through PKIKP waves, while the second dataset is sensitive to the Earth’s mantle only. However, PcP waves sample relatively long portions (longer than 1000 km) of the LMM in a similar way to PKPab waves. The characteristic length of sampling in the LMM is similar for both datasets. Therefore, we would expect that these two datasets yield a similar result, at least for low-degree LMM structure given the differences in sampling, which would prove that they “see” the LMM in the same way. The resulting harmonic degrees 1 and 2 in the tomographic maps derived from these two datasets are similar (compare Suppl. Fig. 1A-B for harmonic degree 1 and Suppl. Fig. C-D for harmonic degree 2). This argues that the P-wave LMM structure is robustly imaged from two independent datasets.

The similarity of maps representing degree 1 and degree 2 structure illustrates that the CMB topography with scale lengths corresponding to harmonic degrees 1 and 2 does not have a dominant impact on travel times of these two datasets. More specifically, if the CMB topography had a strong degree 1 or degree 2 signal dominating over the velocity heterogeneity, it would generate PKPab-PKIKP and PcP-P differential travel times of opposite sign and the two corresponding tomographic models would have velocity anomalies negatively correlated. This hypothetical effect on travel times is illustrated in the Suppl. Fig. 1E. We do not observe an opposite sign of anomalies, thus we rule out significant long wavelength CMB topography and argue that the trade off between topography and heterogeneity in our tomographic method is minimal. It should be noted however that short scale topography could still affect the travel time residuals, but more waveform data of high quality (and more complete coverage of the LMM) is needed to reach more definitive conclusions.
